# Upregulation of TRPV2 exacerbates age-related hearing loss by promoting oxidative stress in spiral ganglion neurons

**DOI:** 10.1186/s13041-026-01286-2

**Published:** 2026-03-06

**Authors:** Zhidong Zhang, Huan Yin, Huan Cao, Miaomiao An, Yanan Li, Jianwang Yang, Tao Liu, Jiantao Wang, Lei Zhao, Chen Wang, Ruoxiang Miao, Baoshan Wang

**Affiliations:** Department of Otolaryngology-Head and Neck Surgery, The Second Hospital of Hebei Medical University, Shijiazhuang, China

**Keywords:** Age-related hearing loss, TRPV2, Oxidative stress, Spiral ganglion neurons

## Abstract

Age-related hearing loss (ARHL) is a prevalent neurodegenerative condition characterized by the progressive loss of spiral ganglion neurons (SGNs). Although oxidative stress is recognized as a central pathogenic driver of ARHL, the precise molecular triggers that initiate and amplify SGN damage remain elusive. Here, we investigated the role of the Transient Receptor Potential Vanilloid 2 (TRPV2) channel in ARHL. We found that TRPV2 expression was significantly upregulated in the SGNs of aged mice, which was associated with elevated oxidative stress. Pharmacological activation of TRPV2 in 6-month-old mice (a pre-senescent stage with preserved baseline hearing) accelerated the onset of high-frequency hearing damage, as evidenced by auditory brainstem response (ABR) measurements. Consistently, TRPV2 activation exacerbated oxidative damage (assessed by 4-HNE staining) and increased apoptotic cell death (detected via TUNEL) in the SGN population. In primary SGN cultures, TRPV2 overexpression aggravated oxidative stress, whereas TRPV2 knockdown in SH-SY5Y cells (a human neuroblastoma cell line) markedly mitigated the oxidative injury as reflected by reduced 4-HNE. Our findings establish that the age-related upregulation of TRPV2 sensitizes SGNs to oxidative stress, thereby promoting neuronal damage and exacerbating ARHL. This work highlights TRPV2 as a promising therapeutic target for intervening in the progression of ARHL.

**Supplementary Information:**

The online version contains supplementary material available at 10.1186/s13041-026-01286-2.

## Introduction

Age-related hearing loss (ARHL), also known as presbycusis, is hearing loss that occurs along with the aging process. ARHL presents as progressive, bilateral, and symmetrical sensorineural hearing loss [1]. As the population ages, the number of elderly individuals affected by hearing loss is steadily increasing [2]. Elderly patients with hearing loss not only have difficulty communicating with the outside world, but may also suffer from cognitive impairment when left untreated over the long term, severely impacting their quality of life [3].

The cochlear spiral ganglion neuron (SGN) is a bipolar ganglion cell and serves as the first neuron in the auditory transduction pathway. SGNs transfer complex acoustic information from hair cells to the second-order sensory neurons in the cochlear nucleus, where sound processing occurs. One of the mechanisms underlying spiral ganglion cell damage is aging [4]. Given that spiral ganglion cells are non-renewable, their damage often results in irreversible hearing loss, posing significant challenges to the treatment of sensorineural hearing loss (SNHL) caused by SGN damage [5]. Existing evidence indicates that oxidative stress is implicated in SGN damage in ARHL, representing one of the key mechanisms underlying this pathology [6].

The damage to SGNs triggered by oxidative stress is ultimately executed through specific molecular pathways [7]. Among these, the dysregulation of ion channels, which are critical for maintaining neuronal excitability and cellular homeostasis, has been increasingly implicated in the pathogenesis of ARHL [8]. In particular, the Transient Receptor Potential (TRP) channel family, known for its sensitivity to a variety of physical and chemical stimuli including oxidative stress, has emerged as a key player in auditory pathology [9]. However, the roles of other TRP channels, especially TRPV2, in the context of age-related, oxidative stress-induced SGN degeneration remain largely unexplored. Given the involvement of TRPV2 in various neurodegenerative disorders, such as Alzheimer’s disease [10, 11], investigating its role in ARHL is of significant importance.

This study aims to investigate the expression and functional role of TRPV2 in SGNs within an aging model, with the goal of determining whether it contributes to the oxidative damage observed in ARHL and potentially identifying a novel therapeutic target for the management of this condition.

## **Materials and methods**

### Animal

C57BL/6 mice (C57) were obtained from the Charles River Laboratories and housed in the animal breeding center of the experimental platform of Hebei Medical University. All experimental procedures involving animals were approved by the Animal Care and Ethics Committee of Hebei Medical University (No.2022-AE308).

### Auditory brainstem response testing

Auditory brainstem response (ABR) measurements were employed to assess auditory thresholds, as detailed in previous study [12]. Briefly, animals were anesthetized via intraperitoneal injection with 1% pentobarbital sodium prepared in normal saline. Platinum needle electrodes were placed at the midline of the skull (reference electrode), ipsilateral mastoid region (recording electrode), and contralateral hind limb (ground electrode). ABR recordings were obtained using a TDT (Tucker-Davis Technologies) system workstation with SigGen32 software. The hearing threshold was defined as the minimum stimulus intensity required to elicit a reproducible ABR waveform. Testing stimuli included clicks and pure tones with frequencies ranging from 4 to 32 kHz.

### Quantitative real-time polymerase chain reaction

Cochlear tissues or cells were lysed, and total RNA was extracted using an RNA extraction kit (Thermo Fisher Scientific, Waltham, MA, USA) according to the manufacturer’s instructions, with all procedures carried out at 4°C. Subsequently, cDNA was synthesized from the extracted RNA using a reverse transcription kit (Roche, Mannheim, Germany), following the manufacturer’s instructions. Quantitative real-time PCR was performed using a Bio-Rad CFX Connect Real-Time PCR System. The housekeeping gene glyceraldehyde-3-phosphate dehydrogenase (GAPDH) was used as an internal reference, and the relative expression levels were calculated using the 2^−ΔΔCt^ method. The primers were synthesized by Sangon Biotech (Shanghai, China). The primer sequences are as follows: TRPV2 forward primer 5’-GGACCCAAATCGGTTTGACC-3’ and reverse primer 5’-GCGCAGGTACTCTAGCAGTC-3’; GAPDH forward primer 5’-TGTCAGCAATGCATCCTGCA-3’ and reverse primer 5’-CCGTTCAGCTGGGATGAC-3’.

### Immunofluorescence

Immunofluorescence staining was performed on frozen sections of mouse cochleae and in vitro cultures of spiral ganglion neurons (SGNs). The cochlear sections or SGNs were first blocked with 5% bovine serum albumin (BSA) in 0.1% Triton for one hour at room temperature and then incubated with primary antibodies against TRPV2(1:200), Tuj1 (1:200, Biolegend, 801201), 4-HNE (1:200), 3-NT (1:200) or Nrf2(1:50) overnight at 4 °C. After washing with PBS to remove the unbound primary antibodies, the samples were incubated with the corresponding secondary antibodies for one hour at room temperature. Finally, the sections or cells were counterstained with 4′,6-diamidino-2-phenylindole (DAPI) to visualize nuclei.

### TUNEL staining

The cochlear paraffin sections were stained using the kit (Vazyme, #A112, Nanjing, China) with all procedures strictly adhering to the manufacturer’s protocol.

### In vivo administration

Six-month-old C57 mice were randomly divided into two groups: a vehicle control group and a treatment group receiving 100 mg/kg probenecid, a TRPV2 agonist. Probenecid (purchased from MCE, catalog number HY-B0545) was dissolved in a 5% sodium bicarbonate solution and administered via intraperitoneal injection three times per week for one month. The vehicle control group received intraperitoneal injections of an equivalent volume of 5% sodium bicarbonate solvent alone. Hearing function was assessed by recording auditory brainstem responses (ABR) following the probenecid treatment regimen. On day 31, the cochleae were collected for assessment of SGN damage.

### Primary culture of SGNs and cell line

Primary cultures of spiral ganglion neurons (SGNs) were established from suckling mouse cochleae as previously described [13]. Briefly, after dissection, the cochleae were subjected to enzymatic dissociation with 0.25% trypsin. The dissociated cells were then resuspended in a medium supplemented with brain-derived neurotrophic factor (BDNF), and maintained under standard culture conditions (37 °C, 5% CO₂). The human neuroblastoma cell line SH-SY5Y was purchased from Ethephon Biotechnology (Shanghai, China). SH-SY5Y cells were treated with 300 mmol/L D-galactose for 24 h to induce oxidative stress. These cells were cultured under the previously specified conditions [14].

### Cell transfection

The TRPV2 overexpression plasmid (NM_011706) was purchased from Youbio (Changsha, China). For TRPV2 knockdown, control siRNA and TRPV2 siRNA were obtained from Hanbio Biotechnology Co., Ltd. (Shanghai, China). Transfections were performed using Lipomaster 2000 Transfection Reagent (Vazyme, #TL201) according to the manufacturer’s instructions.

### Western blot analysis

Western blot analysis was performed using total protein extracts from cultured cells. Briefly, cells were lysed using RIPA lysis buffer supplemented with PMSF. The protein concentration was determined using a BCA protein assay kit. Equal amounts of protein (30 µg) were mixed with SDS loading buffer, separated by SDS-PAGE gels, and transferred onto polyvinylidene difluoride (PVDF) membranes. After blocking with 5% non-fat milk in TBST, the membranes were incubated overnight at 4 °C with the following primary antibodies: anti-4-HNE (1:5000), anti-3-NT (1:1000), anti-TRPV2 (1:500), and anti-GAPDH (1:5000). Following primary antibody incubation, membranes were incubated with appropriate horseradish peroxidase-conjugated secondary antibodies for 1 h at room temperature. Protein bands were detected using an enhanced chemiluminescence (ECL) detection system.

### Statistical analysis

All data were presented as the mean ± SEM. Statistical analyses were performed using GraphPad Prism version 9. For comparisons between two groups, an unpaired Student’s t-test was used. For comparisons among multiple groups, one-way analysis of variance (ANOVA) was performed, followed by Bonferroni post hoc test. A p value of less than 0.05 was considered statistically significant.

## Results

### Mice with ARHL exhibit elevated TRPV2 expression in their spiral ganglion neurons

**Fig. 1:**
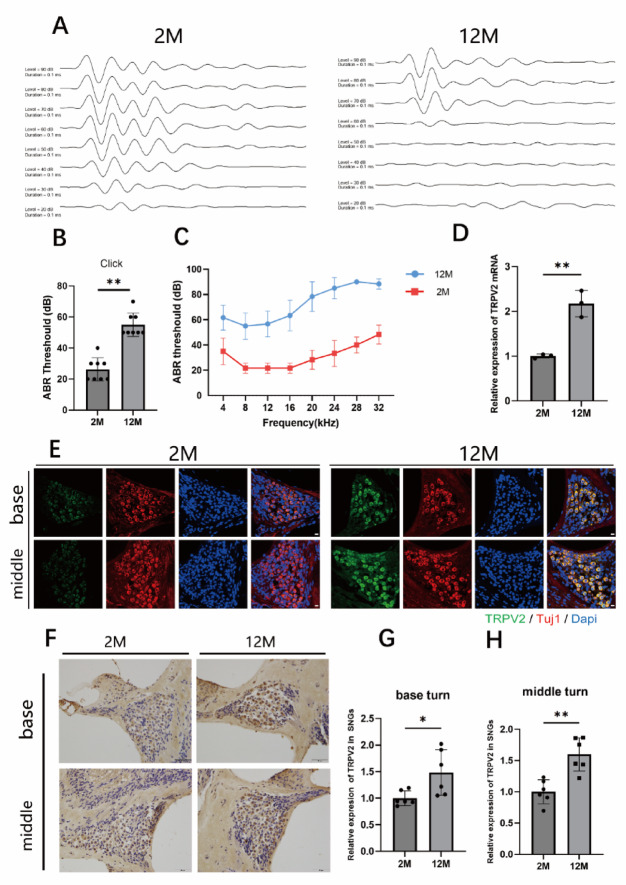
TRPV2 expression is upregulated in the spiral ganglion neurons of aged mice with age-related hearing loss. **A** Representative auditory brainstem response (ABR) waveforms to click stimuli in 2-month-old (2 M) and 12-month-old (12 M) mice. **B** Statistical analysis of ABR thresholds in response to click stimuli. **C** ABR thresholds in response to pure-tone stimuli (4–32 kHz) in 2 M and 12 M mice. **D** Quantitative real-time PCR analysis of TRPV2 mRNA expression in the cochlea of 2 M and 12 M mice. **E** Confocal images showing immunofluorescence staining of TUJ1 (red) and TRPV2 (green) in the basal and middle turns of the spiral ganglion (scale bar: 10 μm). **F** Immunohistochemical staining of TRPV2 in the cochlea (scale bar: 50 μm). **G**, **H** Statistical analysis of TRPV2 expression. Data are presented as mean ± SEM (*n* = 6 per group). *p < 0.05, **p < 0.01 vs. 2 M (unpaired t-test)

To investigate the contribution of TRPV2 to ARHL, we first measured ABR in C57 mice at the age of 2-month-old and 12-month-old to assess hearing function (Fig. 1A–C). ABR recordings revealed a pronounced frequency-dependent elevation of thresholds in 12-month-old mice, compared with 2-month-old mice. The ABR threshold in response to click stimuli was significantly elevated in 12-month-old mice (*p* < 0.0001; Fig. 1B). Consequently, 12-month-old C57 mice were adopted as an ARHL model, while 2-month-old mice served as young controls.

We used real-time PCR to assess the expression levels of TRPV2 in the cochlea. Our results revealed that cochlear TRPV2 levels were higher in mice at 12 months of age (*p* = 0.0024; Fig. 1D), which correlated with elevated hearing thresholds. Subsequently, we examined TRPV2 expression in spiral ganglion neurons. Immunofluorescence staining showed that TRPV2 was present in spiral ganglion neurons from both young and ARHL mice. Moreover, a more intense TRPV2 fluorescence was present in SGNs from ARHL mice compared with those from young mice. Immunohistochemical analysis further indicated that the expression of TRPV2 in SGNs of ARHL mice was increased in both the basal (*p* = 0.0253) and middle (*p* = 0.0012) turns of the cochlea (Fig. 1E–H).

### Increased oxidative damage to SGNs in ARHL mice

**Fig. 2:**
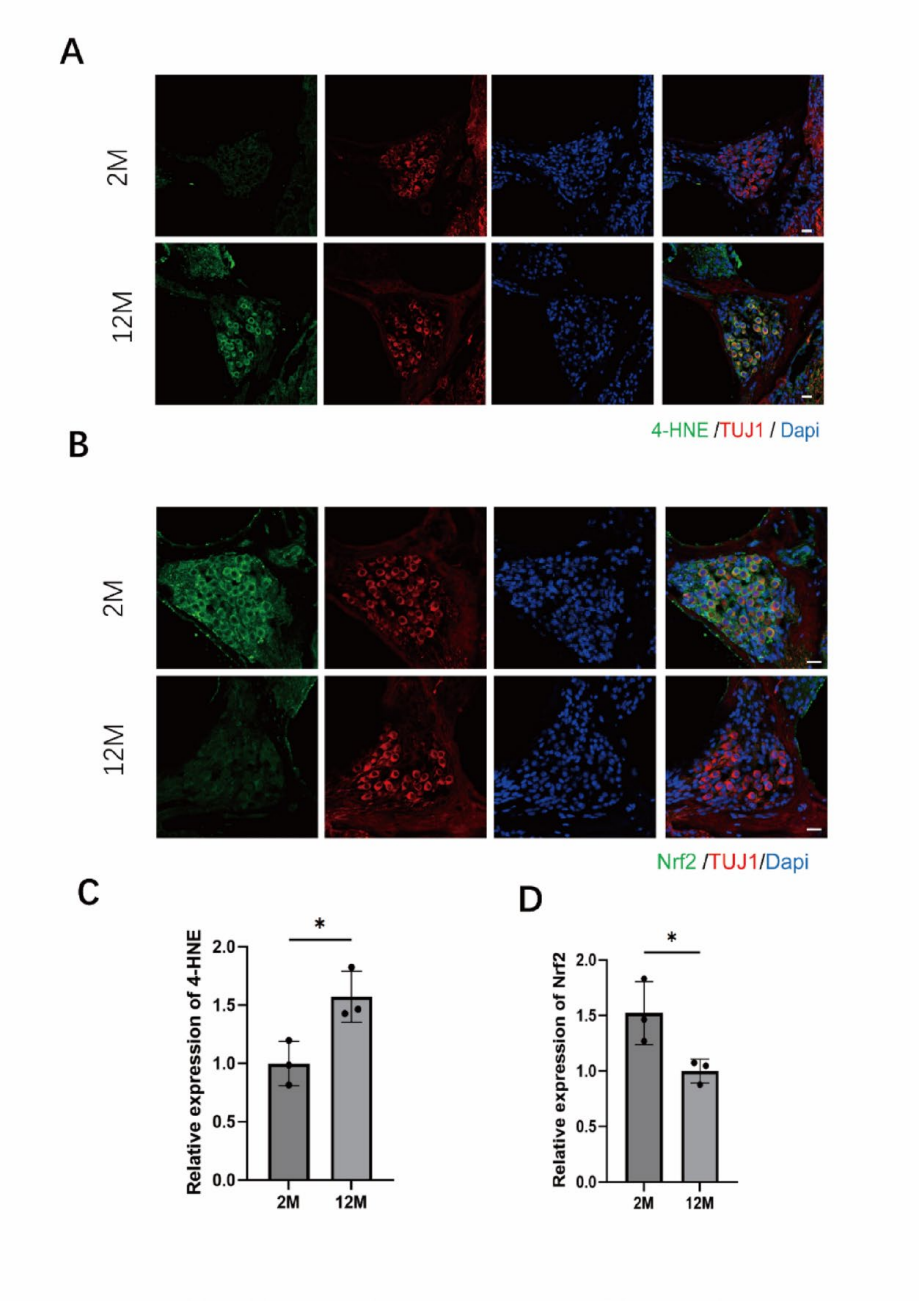
Increased oxidative stress and impaired antioxidant response in spiral ganglion neurons of aged mice. **A** Representative confocal images showing immunofluorescence staining of 4-hydroxynonenal (4-HNE) in spiral ganglion neurons from 2-month-old (2 M) and 12-month-old (12 M) mice (scale bar: 20 μm). **B** Representative confocal images showing immunofluorescence staining of the transcription factor Nrf2 in spiral ganglion neurons from 2 M and 12 M mice (scale bar: 20 μm). **C** Quantitative analysis of 4-HNE fluorescence intensity. **D** Quantitative analysis of Nrf2 fluorescence intensity. Data are presented as mean ± SEM (*n* = 6 per group). *p < 0.05 vs. 2 M (unpaired t-test)

To investigate the role of oxidative stress in hearing damage of 12-month-old mice with ARHL, we examined the accumulation of 4-hydroxynonenal (4-HNE), a well-known marker of oxidative stress, in SGNs using immunofluorescence. The results revealed intense 4-HNE staining in SGNs of ARHL mice, indicating that oxidative stress is a key factor in the damage to these neurons (Fig. 2A, C). Concurrently, we assessed the expression of transcription factor NF-E2-related factor (Nrf2), a critical regulator of the cellular response to oxidative stress. We found that Nrf2 expression was significantly decreased in SGNs of ARHL mice (*p* = 0.0411) (Fig. 2B, D). This downregulation suggests impaired activation of the Nrf2 signaling pathway, which may contribute to increased oxidative stress injury to SGNs in ARHL mice.

### TRPV2 activation exacerbates SGN oxidative stress and hearing loss in mice

**Fig. 3:**
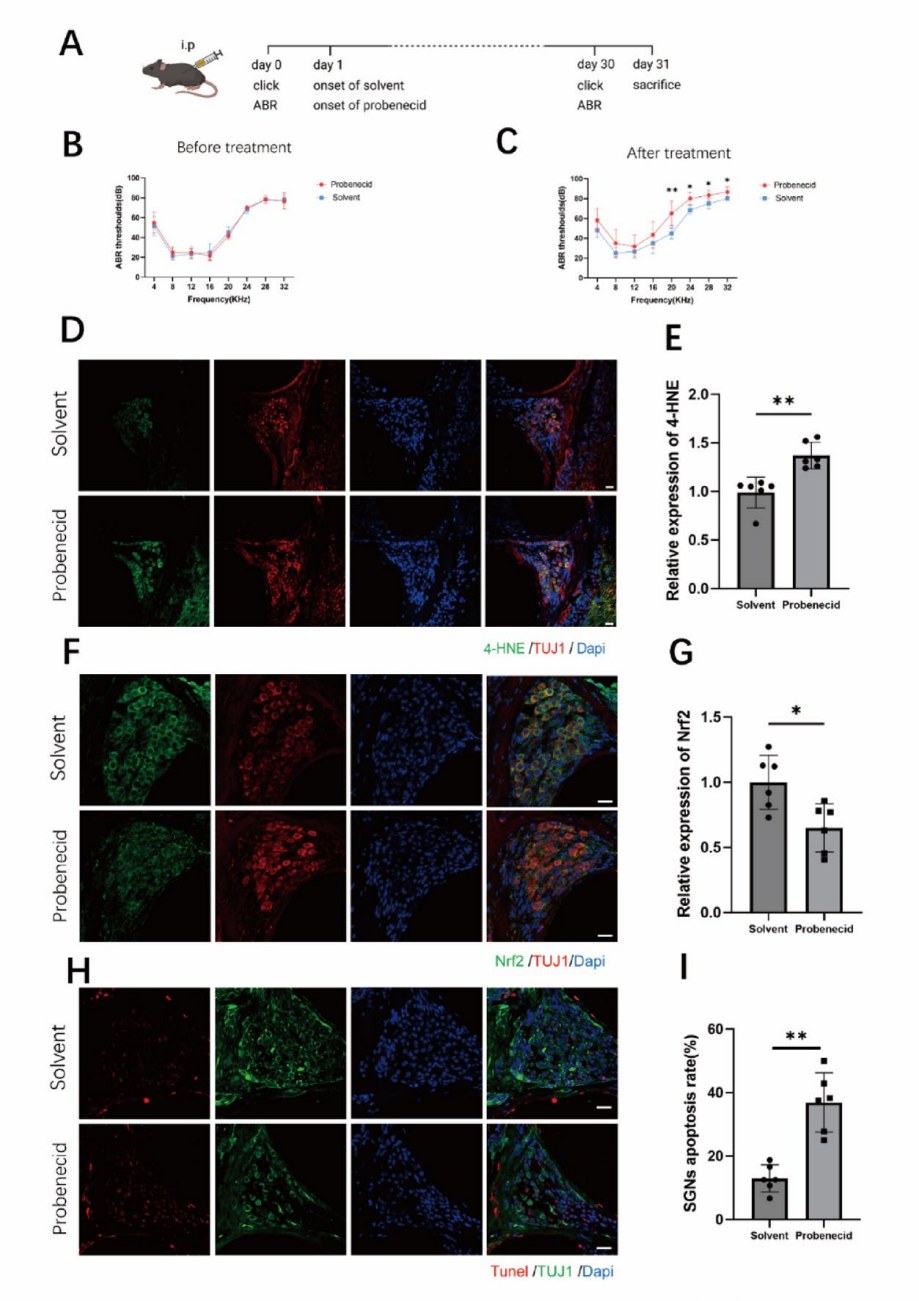
Pharmacological activation of TRPV2 exacerbates hearing loss and oxidative stress, impairs the antioxidant response, and induces apoptosis in spiral ganglion neurons. **A** Schematic diagram illustrating the experimental timeline for in vivo administration of the TRPV2 agonist probenecid (100 mg/kg) or vehicle control in 6-month-old C57BL/6 mice. **B** Representative auditory brainstem response (ABR) waveforms from the vehicle control and probenecid-treated groups. **C** Statistical analysis of ABR thresholds for the solvent control group and probenecid-treated groups (*n* = 6 per group). **D**, **E** Representative confocal images showing immunofluorescence staining of 4-HNE (green) and the neuronal marker TUJ1 (red) in spiral ganglion neurons from the vehicle control and probenecid-treated groups (scale bar: 20 μm). **F**, **G** Representative confocal images showing immunofluorescence staining of Nrf2 (green) and TUJ1 (red) in spiral ganglion neurons from the vehicle control and probenecid-treated groups (scale bar: 20 μm). **H**, **I** TUNEL staining (red) showing apoptotic cells in the spiral ganglion of the vehicle control and probenecid-treated groups. Neurons are labeled with TUJ1 (green), and cell nuclei are counterstained with DAPI (blue). Data are presented as mean ± SEM (*n* = 6 per group), *p < 0.05 (unpaired t-test)

Adult C57 mice began to exhibit hearing loss at 6 months of age. To investigate whether activating TRPV2 would exacerbate hearing loss and damage to SGNs, we divided the mice with uniform hearing into two groups: a solvent control group and a TRPV2 agonist group. The TRPV2 agonist group received intraperitoneal injections of probenecid (100 mg/kg), a specific agonist of TRPV2, three times per week for one month. After this period, we measured the ABRs of both groups and collected samples. The results showed an increased ABR threshold in the frequency of 20 kHz (*p* = 0.0045), 24 kHz (*p* = 0.0014), 28 kHz (*p* = 0.0219), 32 kHz (*p* = 0.0493) for the agonist group compared with the control group (Fig. 3C), indicating a decline in hearing function in the probenecid-treated group following TRPV2 activation.

Immunofluorescence analysis showed that the 4-HNE fluorescence intensity in the agonist group was significantly higher than that in the solvent control group (*p* = 0.0012; Fig. 3D, E), indicating increased oxidative stress. Additionally, more apoptosis was observed in the SGNs of the agonist group (*p* = 0.0002; Fig. 3H, I). Meanwhile, the fluorescence intensity of Nrf2 was decreased in the agonist group (*p* = 0.0117; Fig. 3F, G), indicating that the TRPV2 agonist probenecid exacerbated SGN oxidative stress damage.

### In vitro validation of TRPV2 function in SGN oxidative stress

**Fig. 4:**
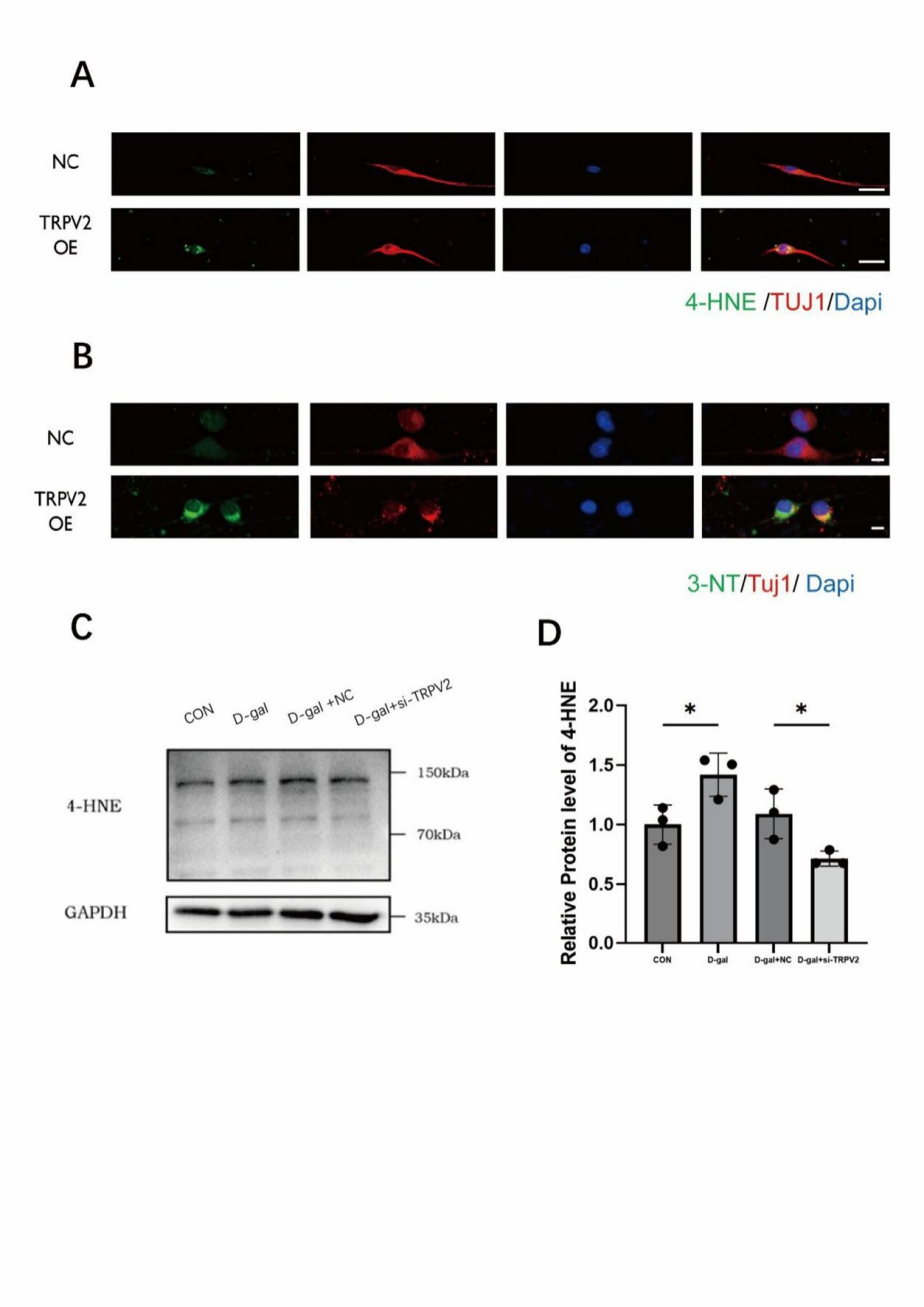
TRPV2 overexpression exacerbates oxidative stress in primary SGNs, while its knockdown attenuates oxidative injury in SH-SY5Y cells. **A** Representative immunofluorescence images showing 4-HNE (green) and the neuronal marker TUJ1 (red) in primary cultured spiral ganglion neurons (SGNs) transfected with either empty vector pcDNA3.1 (Negative Control, NC) or a TRPV2 overexpression plasmid (OE). Scale bar: 20 μm. **B** Representative immunofluorescence images showing 3-NT (green) and TUJ1 (red) in primary cultured SGNs from the same experimental groups. Scale bar: 20 μm. **C**, **D** Western blot analysis of 4-HNE protein expression in SH-SY5Y cells under D-galactose-induced oxidative stress, following transfection with control siRNA (CON) or TRPV2-targeting siRNA. Data are presented as mean ± SEM (*n* = 3 per group). *p < 0.05 (one-way ANOVA with Bonferroni’s post hoc test)

To further investigate the link between TRPV2 upregulation and spiral ganglion neuron (SGN) damage, we cultured primary SGNs and utilized a plasmid to overexpress TRPV2 in these cultured neurons. We then assessed the expression levels of 4-HNE and 3-nitrotyrosine (3-NT) using immunofluorescence (Fig. 4A, B). In comparison with the negative control (NC) group, the fluorescence intensity of 4-HNE and 3-NT was markedly enhanced in TRPV2 overexpression group. This suggests that TRPV2 may serve as a conduit to exacerbate oxidative stress-induced damage in SGNs.

Additionally, we employed human neuroblastoma SH-SY5Y cells as a model system to establish an oxidative stress injury model using 300 mmol/L D-galactose. Subsequently, we used small interfering RNA (siRNA) to knockdown TRPV2 expression in SH-SY5Y cells (*p* < 0.0001; Figure S1 A) to determine whether this intervention could mitigate the cell damage caused by D-galactose treatment, thereby assessing the role of TRPV2 in oxidative stress. TRPV2 was upregulated after the D-galactose treatment (*p* = 0.0337), and could be knocked down by TRPV2 siRNA (*p* = 0.0357; Figure S1 B, C). Western blot analysis showed that following D-galactose treatment, there was an increase in the expression of 4-HNE (*p* = 0.0279), while knockdown of TRPV2 significantly reduced 4-HNE levels compared with the D-galactose treatment group (*p* = 0.0452; Fig. 4C, D). This indicates that TRPV2 knockdown effectively ameliorated the oxidative stress damage caused by D-galactose.

## Discussion

The spiral ganglion neurons (SGNs) play a crucial role in the transmission of auditory signals. Given the non-renewable nature of SGNs, treating sensorineural hearing loss (SNHL) that results from their damage presents significant challenges. SGN damage is recognized as a critical mechanism of ARHL [15]. The dysregulation of various types of ion channels, including non-selective TRP channels, potassium channels, and calcium channels in SGNs is closely related to this damage [9, 16]. Recent research indicates that the upregulation of calcium ion channels in SGNs of ARHL mice may exacerbate the apoptosis of SGNs [17]. Accumulating evidence underscores the expression and functional involvement of multiple Transient Receptor Potential Vanilloid (TRPV) channel subtypes in the auditory system, where they contribute to essential processes such as mechanotransduction, ionic homeostasis, and cellular stress responses. However, their distinct and often divergent roles in the pathogenesis of age-related hearing loss (ARHL) are increasingly recognized. Several TRPV members appear to exert protective functions: genetic ablation of TRPV3 in mice causes progressive hearing loss [18], and TRPV5/6 downregulation contributes to degenerative cascades [19]. In contrast, TRPV1, while essential for auditory function [20], can facilitate aminoglycoside uptake and exacerbate SGN damage [21]. Our study focuses on TRPV2, whose contribution to ARHL remains poorly understood.

In this study, we examined the expression levels of TRPV2 in SGNs from both ARHL and young mice, revealing a significant upregulation of TRPV2 in the SGNs of ARHL mice. Our findings underscore the detrimental role of TRPV2 in auditory health, as we demonstrated that both the activation and overexpression of TRPV2 exacerbate the damage to SGNs. Conversely, the knockdown of TRPV2 was shown to mitigate oxidative stress-induced damage in aged mice.

We observed that TRPV2 expression in SGNs of 12-month-old C57 mice with ARHL was greatly upregulated as determined by immunofluorescence and immunohistochemistry staining. These results indicated a potential link between TRPV2 and ARHL. Previous studies have shown that oxidation can sensitize and activate TRPV2 [22]. Therefore, to further investigate the role of TRPV2 upregulation in aging SGNs, we measured oxidative stress levels in SGNs of ARHL mice.

Interestingly, our study also revealed an increase in oxidative stress that correlated with the upregulation of TRPV2 in SGNs. 4-HNE (4-hydroxynonenal), a byproduct of lipid peroxidation, is formed when polyunsaturated fatty acids (PUFAs) undergo free radical chain reactions under oxidative stress conditions [23]. By measuring 4-HNE expression in SGNs, we can assess the extent of oxidative stress damage to these neurons. Our observations indicated a significant rise in 4-HNE expression in the SGNs of ARHL mice, suggesting oxidative injury in aging SGNs.

Nuclear factor erythroid-2-related factor 2 (Nrf2) is a pivotal regulator that helps cells combat oxidative stress and is involved in mitigating oxidative stress damage in various types of hearing loss [24]. The progression of ARHL is closely linked to the downregulation of Nrf2 [7, 25]. Prior research has demonstrated that Nrf2 knockout mice experience more severe age-related progressive hearing loss than their wild-type counterparts of the same age [26]. Given the widespread expression of Nrf2 in the cochlea, our study evaluated Nrf2 expression in SGNs and discovered a downregulation of Nrf2 in ARHL mice compared to young C57 mice. Collectively, these findings suggest that aging SGNs suffer from exacerbated oxidative stress damage, and their capacity to counteract this damage is diminished.

To further explore the interplay between the elevated TRPV2 expression and oxidative stress, we administered the TRPV2 agonist probenecid to 6-month-old C57 mice, which already exhibited mild age-related high-frequency hearing loss [17]. This intervention was designed to elucidate the role of TRPV2 in oxidative stress-induced damage to spiral ganglion neurons (SGNs). We found that probenecid treatment significantly exacerbated hearing loss in these mice, as evidenced by elevated auditory brainstem response (ABR) thresholds across frequencies ranging from 20 kHz to 32 kHz. Moreover, histological analysis revealed increased oxidative stress markers and apoptosis in the SGNs of probenecid-treated mice compared to controls.

Collectively, our in vivo experiments demonstrate that TRPV2 activation by probenecid potentiates oxidative stress damage and apoptosis in SGNs, thereby worsening hearing loss. These findings underscore the critical role of TRPV2 in mediating oxidative stress-related neuronal damage in the context of age-related hearing loss.

Building on our in vivo findings, we extended our investigation to an in vitro setting to dissect the direct effects of TRPV2 on oxidative stress in SGNs. We cultured primary SGNs from neonatal mice and observed that TRPV2 upregulation alone was sufficient to exacerbate oxidative damage in these neurons. Additionally, we employed the SH-SY5Y cell line, a widely used human neuroblastoma model for neuronal studies [27]. We induced oxidative stress damage in SH-SY5Y cells using D-galactose (D-gal) and knocked down the TRPV2 expression using small interfering RNA (siRNA). Strikingly, TRPV2 knockdown mitigated oxidative stress damage in these cells, highlighting TRPV2 as a potential therapeutic target for oxidative stress-related neuronal injury.

While our study firmly establishes TRPV2 as a key player in oxidative stress-induced damage, the precise downstream molecular mechanisms remain to be elucidated. We did find that Nrf2, a key transcription regulator in the antioxidative stress signaling pathway [28], is implicated in this process. However, this gap is primarily due to the current technical challenges associated with the in vitro study of SGNs, including the lack of an immortalized cell line and the limited yield of primary cells. Future studies utilizing novel SGN-like cell models or advanced in vivo techniques will be crucial to uncover the detailed signaling pathways. These advancements will build on the current findings, which have revealed a novel pathogenic mechanism underlying ARHL and identified TRPV2 as a highly promising therapeutic target for protecting spiral ganglion neurons and preserving auditory function in the aging population.

In conclusion, our findings establish that the age-related upregulation of TRPV2 in SGNs renders them more susceptible to oxidative stress, which in turn promotes neuronal damage and exacerbates ARHL. This study not only reveals a novel pathogenic mechanism underlying ARHL but also identifies TRPV2 as a highly promising therapeutic target for protecting SGNs and preserving auditory function in the aging population. From a clinical perspective, the development of TRPV2 channel inhibitors or small-molecule drugs capable of modulating its function holds promise for providing therapeutic benefits for age-related hearing loss (ARHL). Although no drugs specifically targeting TRPV2 have yet been approved for clinical use, the discovery of lead compounds has demonstrated the feasibility of pharmacological intervention. For instance, the natural product piperlongumine has been identified as a selective allosteric antagonist of TRPV2 [29]. More significantly, in models of neurological diseases, newly discovered TRPV2 inhibitors have demonstrated notable neuroprotective effects in cerebral ischemia-reperfusion injury [30], which indirectly supports their therapeutic potential in degenerative diseases of both central and sensory neurons. Based on this rationale, interventions targeting TRPV2 offer promise for protecting spiral ganglion neurons (SGNs). However, given the non-regenerative nature of SGNs, intervention may need to be initiated in the early stages of hearing loss, prior to irreversible neuronal apoptosis. Inhibiting oxidative stress driven by excessive TRPV2 activity could delay or halt the neurodegenerative process.

## Supplementary Information

Below is the link to the electronic supplementary material.

Supplementary Material 1

Supplementary Material 2

Supplementary Material 3

## Data Availability

The datasets used and/or analyzed during the current study are available from the corresponding author on reasonable request.

## Abbreviations

ARHL: Age-related hearing loss
SGN(s): Spiral ganglion neuron(s)
TRPV2: Transient receptor potential vanilloid 2
ABR: Auditory brainstem response
4-HNE: 4-Hydroxynonenal
PUFA: Polyunsaturated fatty acids
Nrf2: Nuclear factor erythroid-2 related factor 2
GAPDH: Glyceraldehyde-3-phosphate dehydrogenase
BSA: Bovine serum albumin
DAPI: 4′,6-diamidino-2-phenylindole
SNHL: Sensorineural hearing loss
BDNF: Brain-derived neurotrophic factor
siRNA: Small interfering RNA
3-NT: 3-Nitrotyrosine
