# Supplementary figures and images for: Upregulation of TRPV2 exacerbates age-related hearing loss by promoting oxidative stress in spiral ganglion neurons
Source: Mol Brain. 2026 Mar 6;19:24. doi: 10.1186/s13041-026-01286-2 (PMC13077861; doi:10.1186/s13041-026-01286-2)

**immunoblots of Fig. 4C**

Anti-4-HNE


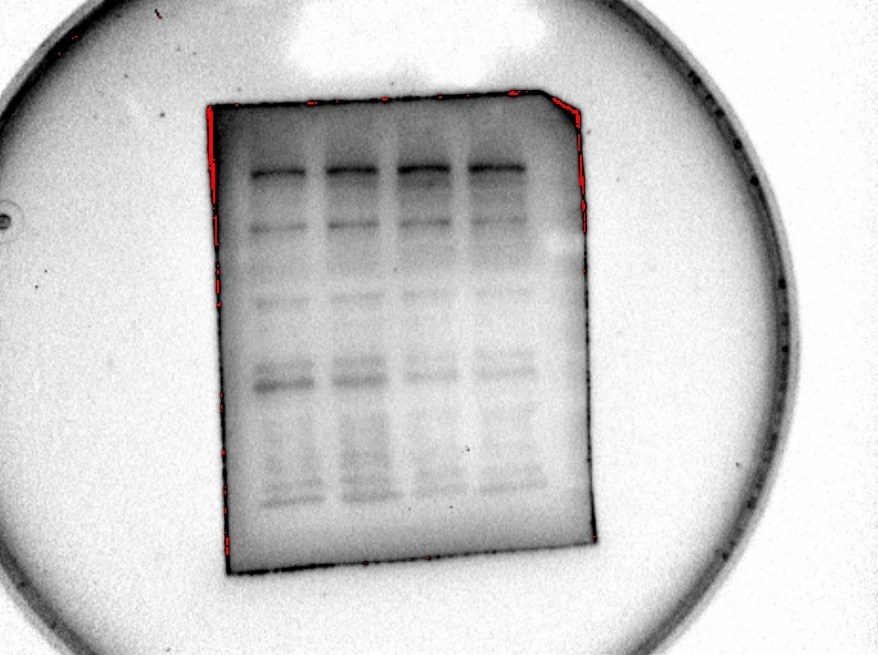


4-HNE


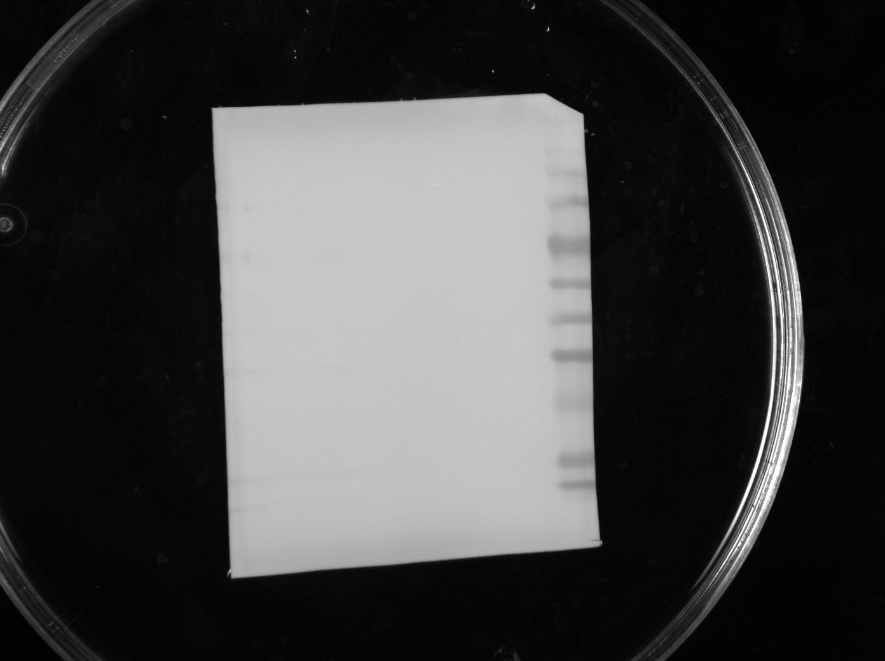


Anti-GAPDH


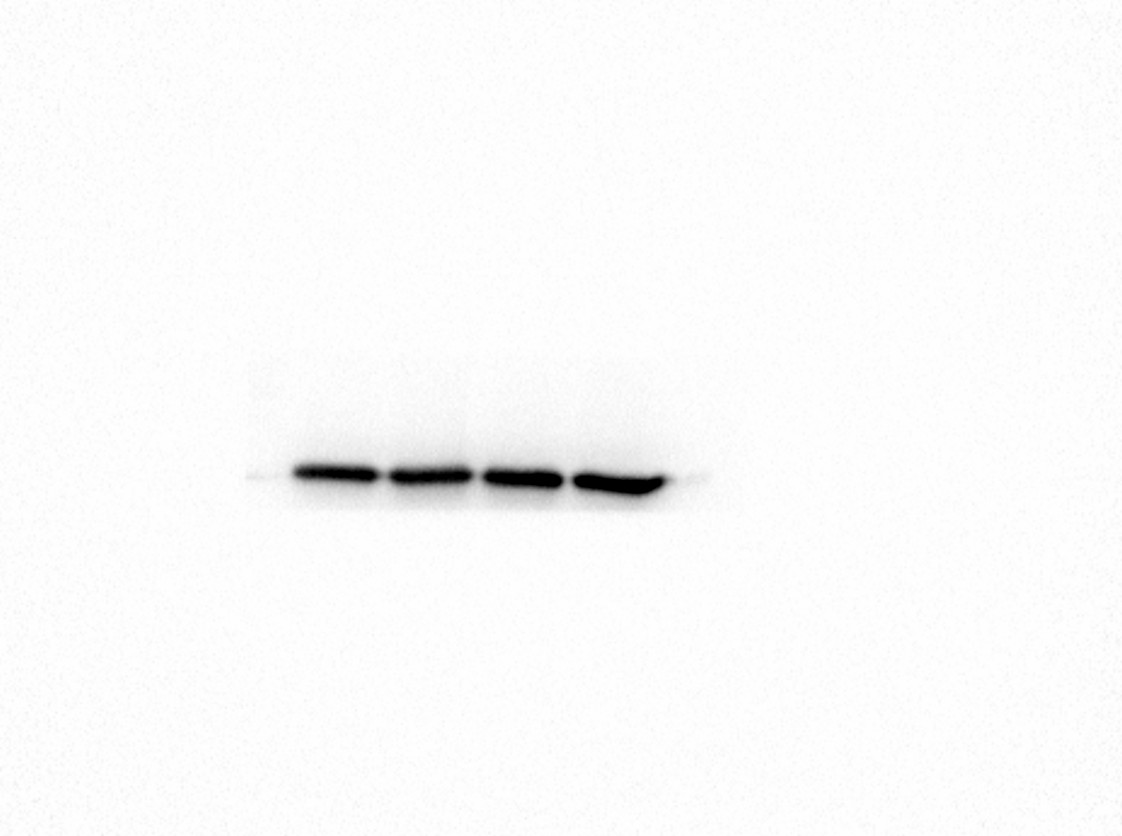


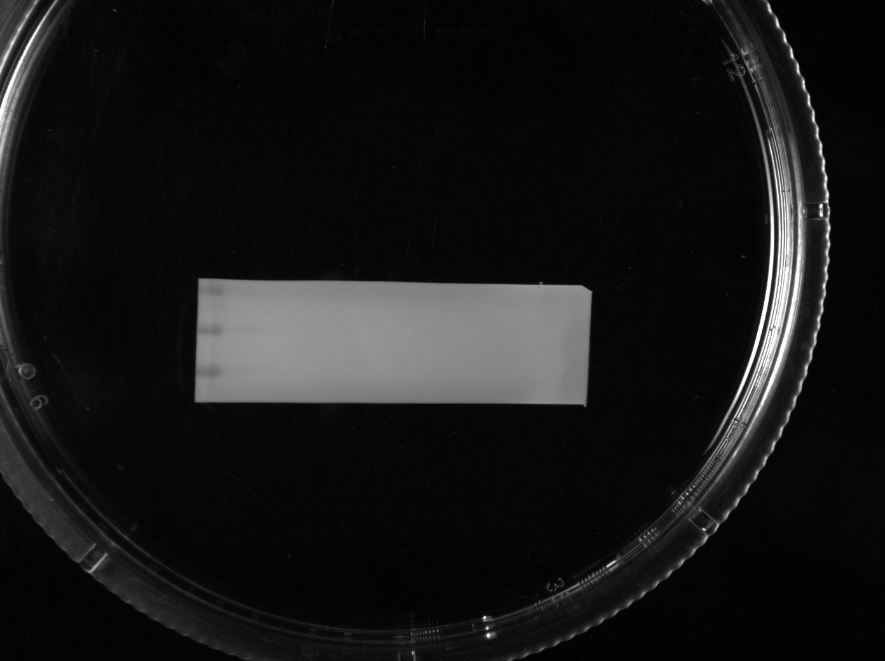


**immunoblots of Fig. S1B**

Anti-TRPV2


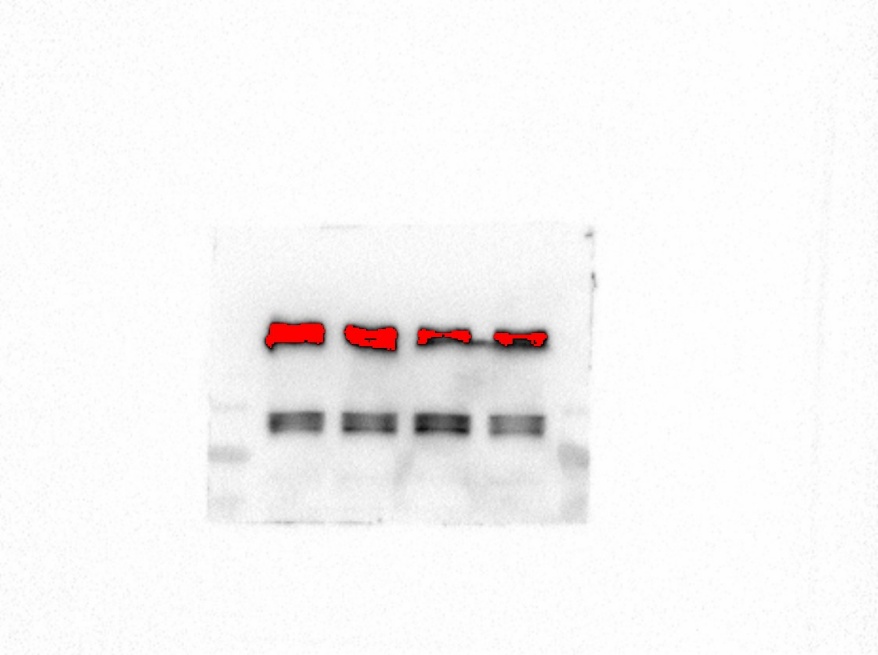


TRPV2


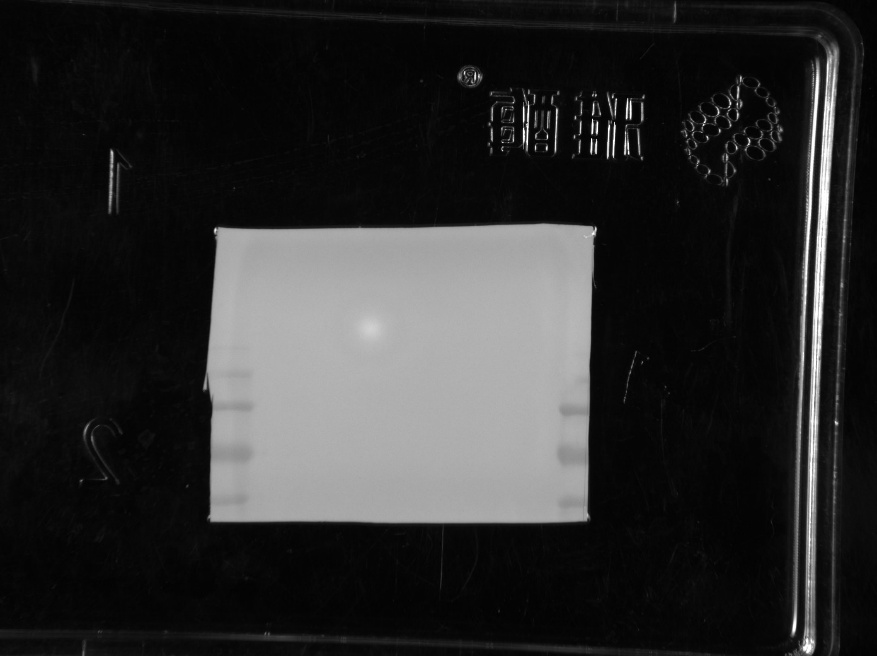


Anti-GAPDH


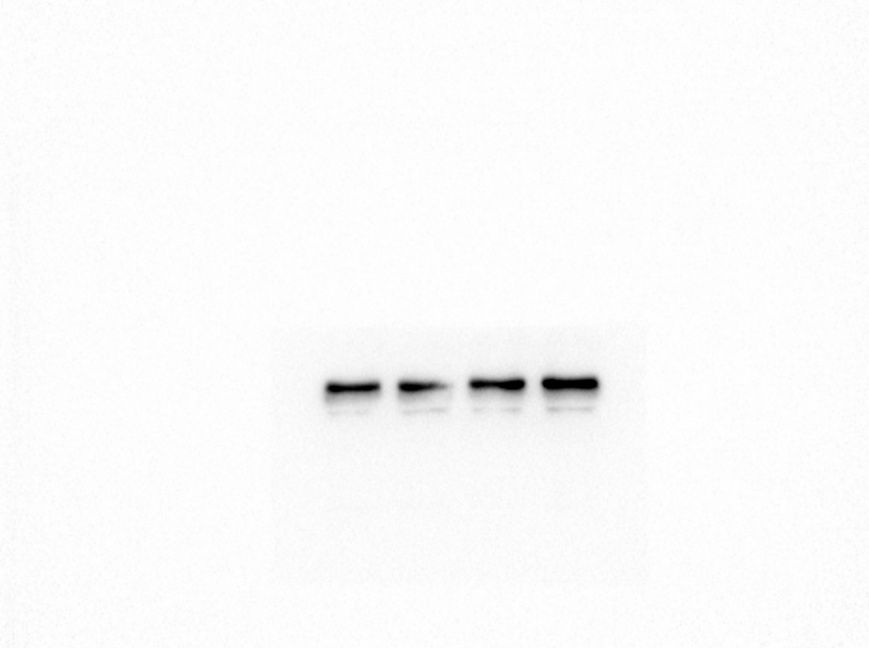


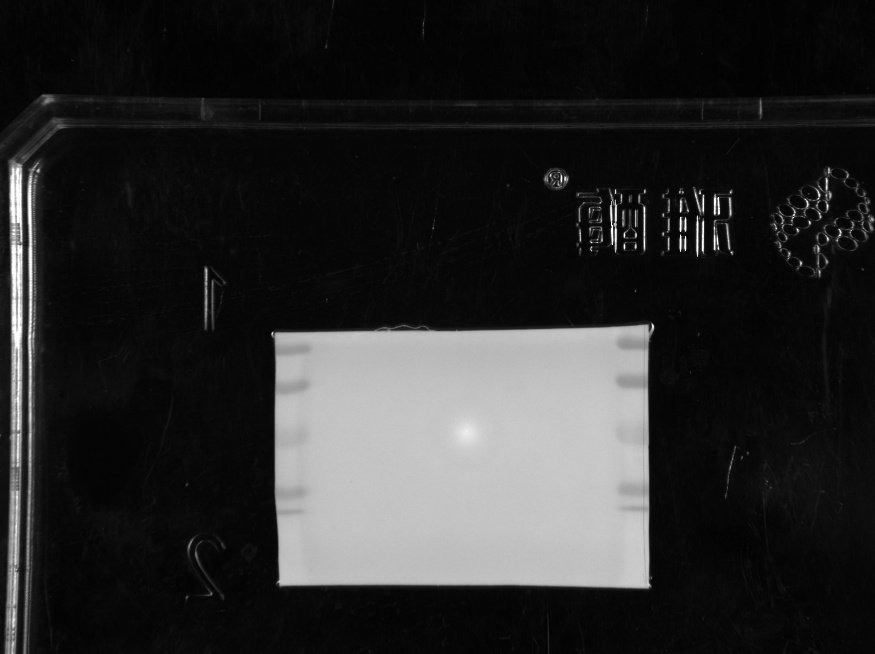


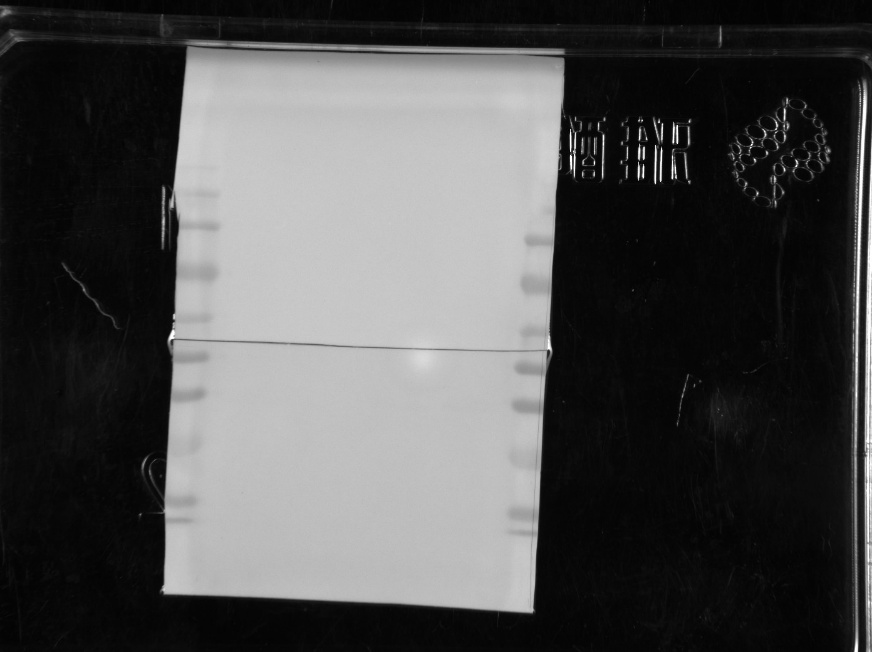

Supplement: Supplementary file 1 — Supplementary Material 1 [file 13041_2026_1286_MOESM1_ESM.docx]
