# Supplementary material for: Upregulation of TRPV2 exacerbates age-related hearing loss by promoting oxidative stress in spiral ganglion neurons
Source: Mol Brain. 2026 Mar 6;19:24. doi: 10.1186/s13041-026-01286-2 (PMC13077861; doi:10.1186/s13041-026-01286-2)
Supplement: Supplementary file 3 — Supplementary Material 3 [file 13041_2026_1286_MOESM3_ESM.docx]

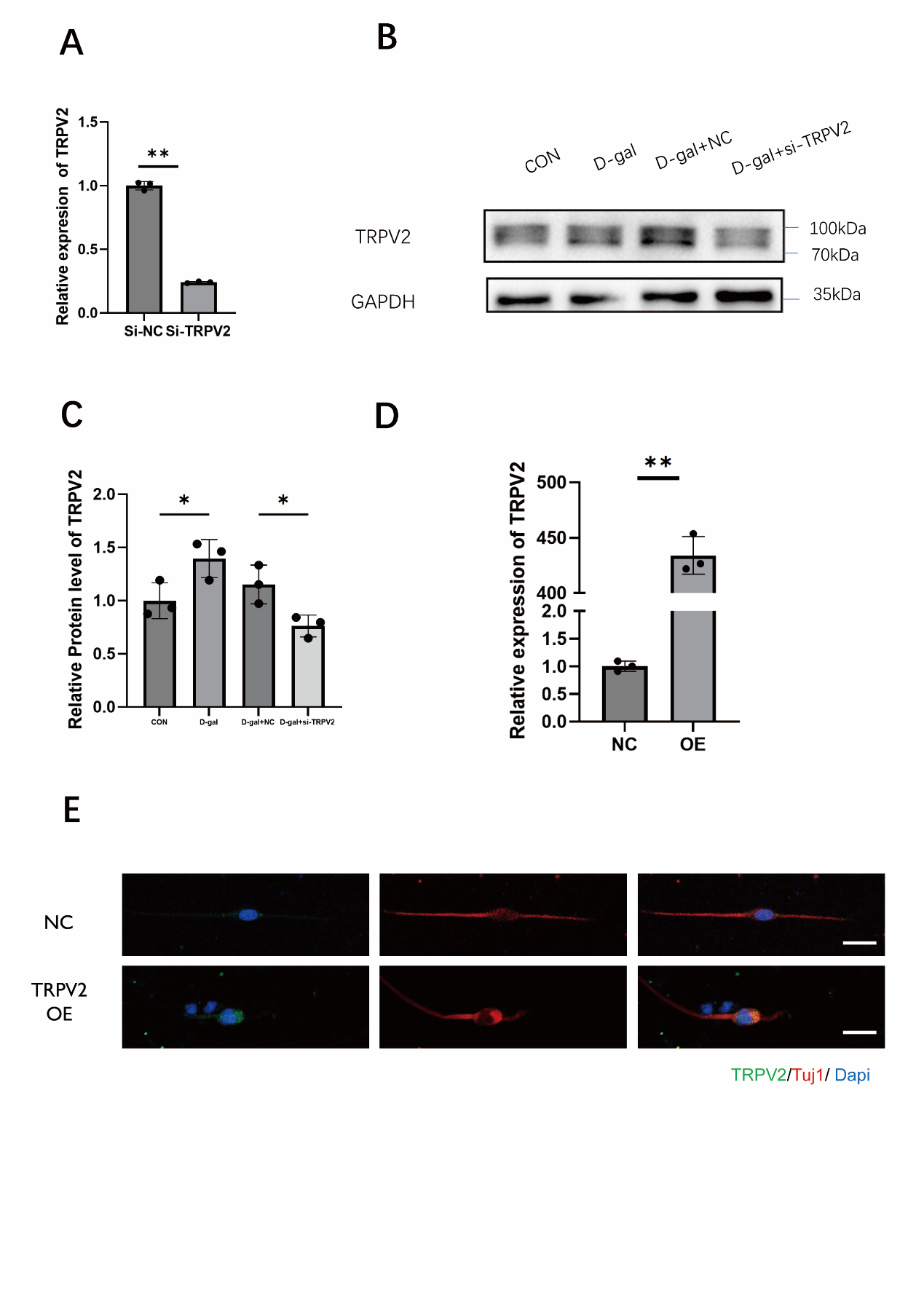


Figure. S1 Validation of TRPV2 knockdown and overexpression efficiency. (A) qPCR analysis confirming the efficient knockdown of TRPV2 mRNA by siRNA in SH-SY5Y cells. Data are presented as mean ± SEM (n=3 per group). **p < 0.01 (unpaired two-tailed Student's t-test). (B) Western blot showing TRPV2 protein levels in four groups of SH-SY5Y cells: untreated control (CON), d‑gal‑treated (D-gal), d‑gal + empty vector (D-gal+NC), and d‑gal + si-TRPV2 (D-gal+si-TRPV2). Results demonstrate successful TRPV2 knockdown at the protein level, as well as upregulation of TRPV2 upon d‑gal treatment. (C) Statistical analysis of the TRPV2 protein expression from (B). Data are presented as mean ± SEM (n = 3 per group). *p < 0.05 (one-way ANOVA with Bonferroni's post hoc test). (D) qPCR validation of TRPV2 overexpression at the mRNA level following plasmid transfection into primary SGNs. Data are presented as mean ± SEM (n=3 per group). **p < 0.01 (unpaired two-tailed Student's t-test). (E) Immunofluorescence images showing enhanced TRPV2 expression (green) in primary SGNs after transfection with a TRPV2-encoding plasmid (OE), compared to the empty vector control (NC). Neurons are labeled with TUJ1 (red), and nuclei are counterstained with DAPI (blue). Scale bar: 20 μm.
